# Animal Models for Assessment of Infection and Inflammation: Contributions to Elucidating the Pathophysiology of Sudden Infant Death Syndrome

**DOI:** 10.3389/fimmu.2015.00137

**Published:** 2015-03-30

**Authors:** Jane Blood-Siegfried

**Affiliations:** ^1^Duke University School of Nursing, Durham, NC, USA

**Keywords:** anaphylaxis, development, hyperthermia, infection, inflammation, nicotine, serotonin, toxin

## Abstract

Sudden infant death syndrome (SIDS) is still not well understood. It is defined as the sudden and unexpected death of an infant without a definitive cause. There are numerous hypotheses about the etiology of SIDS but the exact cause or causes have never been pinpointed. Examination of theoretical pathologies might only be possible in animal models. Development of these models requires consideration of the environmental and/or developmental risk factors often associated with SIDS, as they need to explain how the risk factors could contribute to the cause of death. These models were initially developed in common laboratory animals to test various hypotheses to explain these infant deaths – guinea pig, piglet, mouse, neonatal rabbit, and neonatal rat. Currently, there are growing numbers of researchers using genetically altered animals to examine specific areas of interest. This review describes the different systems and models developed to examine the diverse hypotheses for the cause of SIDS and their potential for defining a causal mechanism or mechanisms.

## Introduction

Sudden infant death syndrome (SIDS) is the most common cause of infant mortality outside of the neonatal period in developed countries (1–3). It is a diagnosis made when every other possible cause can be excluded by the evaluation of all factors including an examination of the death scene, the review of the medical record, and a thorough autopsy (1, 4, 5). The incidence of SIDS is between 1 and 12 months of age with a peak between 2 and 4 months. Even though infants dying of SIDS appear normal and healthy, they often have had a recent mild viral illness that is not associated with the cause of death (1, 2, 6, 7).

Many of the risk factors associated with SIDS parallel those associated with susceptibility to infections. These include prone sleep position, maternal smoking, environmental tobacco smoke exposure, concurrent viral illness, ethnicity, gender, overheating, and drug or alcohol abuse during pregnancy (8–10). With the current decrease in SIDS due to the “Back to Sleep” campaign, the most significant risk factor continues to be maternal smoking during pregnancy (10–17).

Sudden infant death syndrome is most likely caused by several different mechanisms that trigger unexplained deaths. Animal models are important for evaluating the multiple hypotheses of causation and the relationships between: pathology related to sleep position; hyperthermia; hypoxia; reflux; anaphylaxis; infections; toxic exposures; substance abuse; and the role of neurotransmitters in regulation of basic physiologic functions. Animal models offer the ability to evaluate the interaction between different biologic systems in a single experiment.

This review will first outline the models used to assess the role of infection and inflammatory responses in these infant deaths. It will then review how the results of these studies might be applied to determine if inflammation could affect the physiological responses for other hypotheses proposed.

## Inflammation and Infection

Several findings on autopsy of SIDS infants can be difficult to explain, particularly the finding of intrathoracic petechiae and liquid blood in the heart (18, 19). Evidence detected for inflammation has been summarized in the accompanying paper in this volume (Blackwell et al., this volume). These could be associated with efforts to overcome hypoxia, respiratory distress, suffocation, or resuscitation; however, they could also be due to coagulation problems associated with infection and inflammation (20, 21). There are often signs of inflammation and response to infection that are not due to the underlying, pre-existing symptoms. Thymic involution, is observed in more than half of SIDS cases and represents the response to severe stress (22). Thymic involution, intrathoracic petechiae, and liquid blood around the heart are also described in a developmental animal model of SIDS and are similar to the Rambaud results (23).

## Anaphylaxis

Anaphylaxis is a potentially lethal inflammatory response. It was one of the first hypotheses proposed to explain SIDS and was examined by successful development of an animal model (24). Studies were conducted to evaluate allergy associated with anaphylaxis in conscious guinea pigs that had been previously sensitized to cow’s milk (Table 1). After challenge, these animals died quietly with some gasping and then appeared to fall asleep with the heart continuing to beat for a short time. These animals had two findings commonly found on SIDS autopsy, fluid blood around the heart and an empty bladder. Additionally, there was significant histamine release (24). Buckley showed that beta tryptases were elevated in SIDS infants indicating possible mast cell de-granulation prior to death (25). IgE levels were not affected but this is not a mandatory finding for anaphylaxis (25). Challenge from inhaled regurgitated milk in the lungs in a sensitized infant is a plausible scenario.

**Table 1:** **Animal models examining hypotheses to explain SIDS**.

Hypothesis	Animal	Conclusion	Reference
Anaphylaxis	Guinea pig	Animals previously sensitized died quickly when exposed to cow’s milk	(24)
Synergy of infectious agents	Rabbit	IV administration of different common bacterial toxins induced bradycardia, hypotension, and apnea in neonatal rabbits. Similar to endotoxin-induced shock	(26, 27)
	Chick embryo	Toxins from SIDS infants were lethal in combination. Nicotine potentiated the lethal effect	(28, 29)
	Weanling rats	Bacterial isolates from SIDS infants were lethal to rat pups when combined	(30)
	Neonatal rats	Pre-exposure to Influenza A virus caused a lethal response to a sub-lethal dose of endotoxin in rat pups on PN12	(31, 32)
	Mouse	Testing of toxigenic *E. coli* strains from SIDS infants increased mortality	(33)
	Mouse	Pre-exposure to gamma herpes virus and a sub-lethal dose of endotoxin increased fetal loss and reduced litter size	(34, 35)
Hyperthermia	Piglet	Head covering has its primary effect by increasing body temperature in piglets	(36–38)
	Piglet	Febrile piglets have a delayed response to airway obstruction	(39, 40)
Reflux and infection	Rabbit	Simulated reflux-induced apnea	(41)
	Piglet	Elevated body temperature decreased the protective respiratory response to chemoreflex	(42, 43)
Serotonin and inflammation	Serotonin-deficient mouse	Showed an inability to produce a protective autonomic response	(44)
	Mouse	Neonatal mice deficient in serotonin fail to auto-resuscitate during anoxia and die	(45, 46)
Nicotine and infection	Piglet	Nicotine and infection decreased the protective respiratory response to chemoreflex and hypoxia	(47, 48)
Nicotine and serotonin	Baboon	Prenatal nicotine exposure alters autonomic function and control of the heart via changes in the serotonin system	(49)
Nicotine and autonomic control	Rat	Prenatal nicotine exposure decreased auto-resuscitation in response to apnea. This response is directly associated with the effect of nicotine on the development of autonomic function	(50–57)
Development	Rat	On PN12, rat pups exhibit changes in their respiratory responses in the brain stem	(58–60)
	Rat	Lethal response to influenza A and sub-lethal endotoxin challenge in rat pups on PN12	(31, 32, 61)

## Synergy of Infectious Agents

Assessment of the role of infectious agents and their toxins has employed several animal models. Each of these is considered below.

### Rabbit

The response to intravenous injection of six common bacterial toxins was examined in 1–3 kg rabbits. The animals showed cardiac rate slowing, a drop in blood pressure, and apnea with sudden death. It was concluded that bacteria could produce toxins that cause inflammatory responses similar to those associated with endotoxin-induced shock (26) (Table 1). Catecholamine levels did not increase in these animals when the toxins were administered via the gastrointestinal tract; however, increasing doses administered intravenously demonstrated a dose-related increase in catecholamine levels and sudden death. A healthy gastrointestinal tract is not sensitive to these toxins (27).

### Chick embryo

The lethality of toxins obtained from nasopharyngeal preparations from SIDS infants were tested individually and in combination in chick embryos. Enterobacterial and staphylococcal toxins alone were only lethal at high dilutions, however when combined, these same toxins were lethal at much lower concentrations. Both of these strains are found together more often in the nasopharynx of SIDS victims than in healthy infants (28) (Table 1). When nicotine was added at very low concentrations, it further potentiated the lethal action of these bacterial toxins (29).

### Weanling rats

Weanling rats died rapidly without any symptoms prior to death following the injection of nasopharyngeal bacterial isolates obtained from SIDS. These animals had no signs of illness and negligible signs of inflammation in the heart, liver, and lungs (30) (Table 1). When *E. coli* and *S. aureus* were paired, the animals died more rapidly. Again this demonstrated the lethal synergy between different pathogens.

### Neonatal rat

Blood-Siegfried addressed susceptibility associated with developmental stage in relation to the timing of infectious insults. This model used a double insult with a non-lethal strain of influenza A virus and a sub-lethal dose of endotoxin. Animals were given an intranasal dose of the virus on postnatal (PN) day 10. When they were challenged with a sub-lethal dose of endotoxin 2 days after the viral exposure, 70% of the rat pups died within 8–10 h, quietly without significant symptoms. Animals displayed the characteristic intrathoracic petechiae, liquid blood around the heart, thymic involution, and other findings on pathology that have also been found in infants dying of SIDS (23). Older animals and younger animals did not die (31, 32). SIDS occurs between 2 and 4 months of age in human infants. The narrow window of lethality seen in this animal model on PN12 could be due to an increased susceptibility from normal developmental changes in the immune, endocrine system, or autonomic nervous system (31) (Table 1). These animals had an abnormal cytokine profile in response to that normally seen following endotoxin injection (62).

Pilot work suggests that rat pups exposed to nicotine *in utero* exhibited a similar pattern of mortality following endotoxin injection on PN11 or PN12. It was not necessary to pre-infect these animals with influenza. Prenatal nicotine exposure alone appears to be sufficient to alter the neonatal rat’s physiology to make the animal susceptible to a sub-lethal dose of endotoxin. Work in this model is continuing.

### Mice

Pregnant mice were exposed to *E. coli* in the drinking water. One strain of *E. coli* was toxigenic, obtained from SIDS infants; three other strains were from normal infants. Dams exposed to the toxigenic strain had smaller litter sizes and some runting of the pups. Mortality was 18% for the SIDS *E. coli* strain compared to 9% for non-SIDS isolates (33) (Table 1). In a different mouse model, gamma herpes virus plus a non-lethal dose of endotoxin was responsible for a reduction of litter size and fetal loss (34) (Table 1). These studies point to a double hit hypothesis for fetal and neonatal death. A single toxin or infectious insult might not be lethal on its own, but in the right combination, and at the right developmental stage, they trigger a lethal event. The multi-hit hypothesis also explains why the spiral to death can often not be stopped once it has started, even with exhaustive medical care. The process begins long before it is noticeable and treatment can be started.

In all of these models, an infectious insult was lethal to young animals. The number of infectious insults increased lethality with nicotine exposure also acting synergistically with the infectious agent. These data support the premise that maternal smoking, both prenatal and PN, might increase the lethality of a presumably non-lethal infection in a susceptible infant. Inflammatory mediators can affect many of the mechanisms proposed to explain SIDS. The following section assesses these models and comments on how inflammation might contribute to the lethal processes investigated.

## Respiration and Hyperthermia

Prone sleeping position plays a significant role in SIDS risk, not because of its effect on normal oxygen/carbon dioxide-exchange but rather on an increase in temperature. Elevated body temperature and increased toxin production by bacterial isolates in the nasal pharynx might be a contributing factor (33, 63, 64). Data suggest that prone position will exacerbate the consequences of a viral infection because it promotes an optimum temperature to increase the numbers and variety of bacterial species in upper respiratory secretions and stimulates bacterial toxin production (65).

It is well understood that infection affects the control of respiration and temperature. In studies of infants who died from SIDS while on heart monitors, the cardiac and respiratory tracings resembled those of infants with known infections rather than infants succumbing to asphyxia (66). Alteration of protective mechanisms, gasping, arousal, and efforts to restore normal blood pressure and heart rate are likely to be involved (67, 68). SIDS is more common in the winter months; therefore, these infants are usually well covered to keep them warm. Additionally, infants in a prone position tend to retain body temperature if overwrapped, even in cold weather, they risk becoming hyperthermic (69).

In a piglet model, Galland and colleagues (36, 37) tested the hypothesis that O_2_ consumption could be altered in a cold climate when the face was cold and the body remained hot. They found that artificially induced hyperthermia increased REM sleep, a period associated with risk for SIDS (37) (Table 1). This study promoted the hypothesis that the primary effect on mortality was an increased body temperature rather than any changes in O_2_ or CO_2_ gas exchange (38, 70).

In a separate experiment, piglets developed tachycardia with a drop in blood pressure when heated. Their respiratory rate increased to compensate but they developed hypocapnic alkalosis and a metabolic acidosis. In addition, these animals had necropsy findings similar to that seen in SIDS cases, excessive lung hemorrhage, and alveolar edema (39) (Table 1). Febrile piglets were shown to have a delayed protective response to airway obstruction (40). Hyperthermia resulting from infection or prone position might trigger event leading to SIDS.

## Reflux and Infection

Reflux during sleep has been proposed to have a negative effect on the human infant’s ability of the human infant to auto-resuscitate. Simulated reflux in neonatal rabbits induced obstructive, central, and mixed apnea (41); however, the artificial instillation of a mildly acidic compound in a piglet model, stimulated normal protective responses in sedated and naturally sleeping animals (71, 72) (Table 1). Post-mortem lung changes in these piglets showed petechiae, characteristic of SIDS (73).

In a piglet model decerebrated, vagotomized animals were used to evaluate the effects of apnea and hyperthermia in sudden death. When body temperature was elevated, chemoreflex was prolonged (42) through a central nervous mechanism (43) (Table 1). Prenatal nicotine exposure decreased recovery from laryngeal chemoreflex in heated piglets (74). Again there was a connection with respiratory inhibition and hyperthermia.

*Helicobacter pylori* DNA and antigens have been found in post-mortem tissues of SIDS infants. A rat model has been used to determine if a fatal apnea could result from a response to gastroesophageal reflux (75, 76). The results were inconclusive that *H. pylori* are a primary cause of SIDS; however, this model is consistent with infectious challenge (77–79). Recent work by Highet and colleagues on samples from SIDS infants show that the gut microbiome are significantly different than found in control infants and should be explored further as a cause of inflammation (80).

## Serotonin, Depression, and Inflammation

The serotonergic system of the brain stem, an area that controls heart rate and breathing, is a primary focus for the role of the central nervous system in the factors underlying SIDS. The neurotransmitter serotonin appears to be very sensitive to inflammation; in particular, the inflammatory cytokines often found in autopsy in the central nervous system of SIDS infants (81–83). A specific relationship between increased inflammation and decreased serotonin output has been established in studies on depression (84); the use of anti-inflammatory medications improves serotonin function as it improves depression (85). If SIDS is associated with low levels of serotonin in important structures of the brain stem, it is reasonable to examine the hypothesis that inflammation due to infection might be involved.

Genetically altered animals have been used to explore specific pathology in the serotonin system. An over-expression of serotonin auto-receptors in this mouse model leads to death associated with a decrease in serotonin, drop in heart rate, hypothermia, and a failure to initiate protective autonomic responses (44) (Table 1). Neonatal mice deficient in brainstem serotonin have spontaneous bradycardia in room air and fail to auto-resuscitate during episodic anoxia, ultimately dying from an inability to appropriately increase heart rate (45, 46) (Table 1). Inflammatory cytokines decrease levels of serotonin in the brain stem and might lead to death because of an inability to respond appropriately to key triggers SIDS such as hyperthermia, hypoxia, low blood pressure or decreased heart rate.

## Nicotine

Smoking during pregnancy is a primary risk factor for SIDS (49, 86, 87). The risk is fairly low from environmental tobacco exposure (11, 13, 88–90); however, maternal smoking doubles the risk and that increases 3–4 times when the mother smokes more than 10 cigarettes per day (12, 86). Nicotine is considered a major neuroteratogen (91). Nicotine crosses the placenta during pregnancy and could explain why maternal smoking during pregnancy is more harmful on central respiratory mechanisms than PN exposure to environmental tobacco smoke (10, 13, 51). Prenatal nicotine exposure could result in an increase in susceptibility to other risk factors of SIDS in several ways that involve inflammation.

### Nicotine and infection

The synergy between infection and smoking has been examined in a piglet model. When animals are treated with nicotine and endotoxin then exposed to subglottic acidified saline solution are unable to auto-resuscitate and develop prolonged apnea. Nicotine and an inflammatory mediator interleukin-1β have a similar synergistic effect, decreasing the animal’s ability to respond to apnea (47). These experiments demonstrate the synergistic connection between nicotine and inflammation and lethal apnea (92) (Table 1). While Froen and colleagues did not directly examine the role of infection, inflammatory mediators are often used as proxy for inflammation and it is highly likely that infection is involved.

*In vitro* studies on the effects of infection and risk factors such as cigarette smoke on inflammatory responses have identified that these factors can increase inflammation and enhance pro-inflammatory responses (93, 94), these effects need to be tested in animal models in which they also change the autonomic and serotonergic response to infection.

### Nicotine and serotonin

Prenatal exposure of animals to nicotine has been shown to alter the response of the serotonergic system in the newborn by down-regulating receptors important for normal serotonin function (49, 95). This in turn alters normal function and control of the heart through its action on the autonomic nervous system (49) (Table 1). Any process that challenges the cardiovascular system such as overheating, infection, toxins, and low blood sugar levels, increases SIDS risk (64, 96, 97). Nicotine exposure might increase the risk of SIDS by altering how the infant responds to these challenges.

### Nicotine and autonomic control

Investigators have used the rat model to evaluate the effect of prenatal nicotine exposure on the heart (55), brain (54), and respiration (56, 57) (Table 1). Nicotine stimulates the nicotinic acetylcholine receptor and mimics the effects of acetylcholine (98). Chronic exposure of these receptors during critical prenatal development could explain the abnormalities of receptor expression and acetylcholinesterase activity observed in tissues from SIDS infants. These mechanisms are critical for normal autonomic control of the heart.

A strain of rabbits was developed to examine the effect of cardiac muscarinic receptors on autonomic tone. Similar to infants dying from SIDS, these rabbits had over-expression of muscarinic receptors that was most pronounced between the fifth and the seventh week of life, a time of increased mortality in the rabbits (99). Abnormal autonomic development from maternal smoking during pregnancy might result in infants being unable to arouse and respond to a physiologic insult such as a drop in blood pressure, hypoxia, infection, or overheating.

Mechanisms that regulate inflammation also affect vagal nerve activity through the release of acetylcholine. Data suggest an association between heart rate variability, a prime example of normal vagal function, and inflammation (100). Stress can precipitate both depression and promote inflammatory responses through its action on the sympathetic and parasympathetic nervous system (101). All of these processes are so intertwined that evaluating them as a single response negates the complex sets of interactions in the human body and encourages the use of animal models that can mimic interactions between systems.

## Developmental Susceptibility

One key factor in SIDS is the specific timing of susceptibility. By definition, SIDS infants are younger than 12 months of age with most SIDS occurring between 2 and 4 months. There is a peak around 3 months of age. Evaluation of a condition that occurs during such a specific time during the development of multiple interrelated physiologic systems can be quite difficult. Teasing out the combined effects requires the ability to measure multiple interactions at the same time in many organ systems.

### Development of inflammatory responses

The peak age at which SIDS occurs corresponds to a time when infants are dependent on innate responses to infection. A dysregulation of innate inflammatory responses and the developmental change that mature the infant could contribute to the physiological changes leading to these sudden deaths (31, 32, 94, 102, 103). The immune system is beginning to recognize a large number of foreign antigens and developing its own antibody responses while the protective effect of maternal antibodies is waning (104). Small changes in the development of immune responses can turn a minor illness into a lethal event (62). This could explain why breastfeeding is protective for SIDS because breast milk contains biologically active antibodies that reduce colonization by potentially pathogenic bacteria as well as glycoproteins that reduce attachment of organisms to epithelial cells via lectin like adhesins (105, 106).

Male infants succumb to SIDS slightly more frequently than females. Peripheral blood monocytic cells from adult male donors exposed to cigarette smoke extract produced lower levels of some inflammatory cytokines than those from adult female donors. There were significant correlations between levels of pro-inflammatory cytokines and testosterone, but only in females. Testosterone levels in females correspond to those among male infants in the age range at greatest risk of SIDS. There is potentially an effect between cortisol levels and the testosterone surge in male infants during the time of SIDS risk but this needs to be evaluated further (94).

The normalization of the adult day–night temperature pattern occurs around the time of highest SIDS risk (107). Both human infants (108, 109) and rat pups (110, 111) have a nadir of cortisol production during this time of development that overlaps a time when the immune system is responding to new infections and the corresponding cortisol system might not be able to regulate the response. The levels of cortisol suppress pro-inflammatory responses to bacterial antigens during the day, just before and after the switch to diurnal circadian rhythm. The night-time levels before the switch have the same effect; however night-time levels of cortisol after the switch are much lower and do not have a suppressive effect on the immune system. In fact, they can enhance pro-inflammatory responses [(106); Blackwell et al., this volume]. Developmental immaturity at this age could lead to an inability to produce a protective response to an infection.

### Development of the neural system

The maturity of the autonomic nervous system and baroreflex control of the heart in human infants correspond to times of autonomic imbalance (112). This imbalance is influenced by stage of sleep, head covering, temperature, body position, and nicotine exposure, all known risk factors for SIDS (113–116). In 2- to 4-month olds, there is a significant difference in cardiac response to a hypotensive event in quiet sleep. This is in contrast to that seen in adults and newborns where heart rate elevates and is maintained in an elevated stage for several minutes (117). Ledwidge et al. (118) describes a case where a 5-month-old infant died during a sleep study because the heart rate failed to increase in response to a head-up tilt (decrease in blood pressure), even though all other parameters of the sleep study were normal.

### Development and respiration

Developmental changes in 12-day-old rats had an abrupt switch in brain stem expression of a receptor important for stimulating respiratory control. This period of imbalance is transitory but could leave the respiratory system vulnerable to insult such as infection that often causes apnea in infants (58–60, 119) (Table 1). These represent three very important areas of development that occur around the time of unexplained death that could be attributed to a SIDS-like event. This case supports the hypothesis that abnormal autonomic tone might contribute to SIDS deaths.

## Conclusion

Any animal model used to mimic a human condition should reflect the pathophysiology and other parameters that are indicative of that condition. SIDS by definition is a sudden unexplained death that cannot be defined by symptoms or pathology commonly known to cause death. Because of this definition, there are very few clues to build a model on. The models presented here are an attempt to define possible clues on which to build a model with physical and epidemiologic findings of SIDS.

A developmental model like that described by Blood-Siegfried is critical to tease out the effect of inflammatory responses to infection on interactions among physiological systems and risk factors such as tobacco smoke (31, 32, 61, 120) (Table 1). This model not only causes pathological finding similar to those found on autopsy of SIDS infants but also reflects the narrow window of susceptibility not seen in many other models (23).

Further examination using this model could evaluate other possible mechanistic associations:

1) How does cigarette smoke extract affect 11- and 12-day-old rat pups?
2) How does the stress response as measured by catecholamine and corticosterone levels respond to the insult?
3) Do animals that are susceptible to death have specific changes in the circadian rhythms?
4) What is the normal development of autonomic tone and heart rate variability?

This developmental model allows the researcher to examine the effect of age within the context of the development of these systems and the interactions between systems.

We have only begun to tap into the multiple possibilities of connections involved in sudden and unexplained death. SIDS is a complex syndrome and the answer to its cause/s will require development of appropriate animal models to determine how all the parts fit together.

## Conflict of Interest Statement

I have received no payment or services from a third party for any aspect of the submitted work. The developmental model was initially designed during a post-doctoral fellowship at the National Institute of Environmental Health Sciences. Further work was funded by a small grant from Duke University School of Nursing.
